# Dr. Paul Gibier

**Published:** 1900-07

**Authors:** 


Dr. Paul Gibier, of New York, founder and head of the Pasteur Institute, at No. 313 West Twenty-third St., died at midnight on Saturday, June 9, 1900, at his summer home at Suffern, as a result of a fracture of the skull, received by being thrown from his carriage.

Dr. Gibier was born in the Department of André, France, on October 9, 1851. He was early a student at the University of Paris and was a graduate of the medical department. For some time he was assistant professor of comparative medicine in the Paris Museum, and was resident physician in several of the more prominent Paris hospitals.

In 1885, Dr. Gibier was commissioned by the French Government to study the cholera epidemic that was then raging in Spain, and his work in that line procured for him a gold medal from the French Republic. In 1886 he was made a member of the Legion of Honor on account of work done by him in the cholera plague in the South of France. In 1888 he was sent by the French Government to study the yellow fever conditions in Havana and Florida. On his way home he stopped for some time in New York City.

Dr. Gibier returned to this country in 1889, and in 1890 established the Pasteur Institute at No. 313 West Twenty-third Street. He had recently completed a handsome building at Suffern, and was preparing to open a sanitarium there.

Dr. Gibier leaves a wife and nephew in this country, and his mother and a sister in France. The nephew, Dr. George G. Rambaud, will probably succeed to the direction of the institution.

